# Molecular Implication of PP2A and Pin1 in the Alzheimer's Disease Specific Hyperphosphorylation of Tau

**DOI:** 10.1371/journal.pone.0021521

**Published:** 2011-06-23

**Authors:** Isabelle Landrieu, Caroline Smet-Nocca, Laziza Amniai, Justin Vijay Louis, Jean-Michel Wieruszeski, Jozef Goris, Veerle Janssens, Guy Lippens

**Affiliations:** 1 CNRS-UMR 8576, Federative Research Institute IFR 147, University of Lille-North of France, Villeneuve d'Ascq, France; 2 Protein Phosphorylation and Proteomics Group, Molecular Cell Biology Department, Faculty of Medicine of Leuven University, Leuven, Belgium; The University of Sydney, Australia

## Abstract

**Background:**

Tau phosphorylation and dephosphorylation regulate in a poorly understood manner its physiological role of microtubule stabilization, and equally its integration in Alzheimer disease (AD) related fibrils. A specific phospho-pattern will result from the balance between kinases and phosphatases. The heterotrimeric Protein Phosphatase type 2A encompassing regulatory subunit PR55/Bα (PP2A_T55α_) is a major Tau phosphatase *in vivo*, which contributes to its final phosphorylation state. We use NMR spectroscopy to determine the dephosphorylation rates of phospho-Tau by this major brain phosphatase, and present site-specific and kinetic data for the individual sites including the pS202/pT205 AT8 and pT231 AT180 phospho-epitopes.

**Methodology/Principal Findings:**

We demonstrate the importance of the PR55/Bα regulatory subunit of PP2A within this enzymatic process, and show that, unexpectedly, phosphorylation at the pT231 AT180 site negatively interferes with the dephosphorylation of the pS202/pT205 AT8 site. This inhibitory effect can be released by the phosphorylation dependent prolyl *cis*/*trans* isomerase Pin1. Because the stimulatory effect is lost with the dimeric PP2A core enzyme (PP2A_D_) or with a phospho-Tau T231A mutant, we propose that Pin1 regulates the interaction between the PR55/Bα subunit and the AT180 phospho-epitope on Tau.

**Conclusions/Significance:**

Our results show that phosphorylation of T231 (AT180) can negatively influence the dephosphorylation of the pS202/pT205 AT8 epitope, even without an altered PP2A pool. Thus, a priming dephosphorylation of pT231 AT180 is required for efficient PP2A_T55α_-mediated dephosphorylation of pS202/pT205 AT8. The sophisticated interplay between priming mechanisms reported for certain Tau kinases and the one described here for Tau phosphatase PP2A_T55α_ may contribute to the hyperphosphorylation of Tau observed in AD neurons.

## Introduction

Phosphorylation/dephosphorylation of the neuronal microtubule associated protein Tau regulates in a complex manner its capacity to assemble tubulin into microtubules. It is also associated with the presence of pathological fibrils in neurons of AD patients, which are mainly composed of hyperphosphorylated Tau. Monoclonal antibodies such as AT180 and AT8, recognizing respectively the pT231 [1] and pS202/pT205 [2] Tau phospho-motifs, are available for post-mortem diagnostics of the disease progression, and can define the neurofibrillary lesions at different stages of the disease [3]. The spatial hierarchy observed is equally accompanied by a temporal progression of the phosphorylation pattern of Tau. The T231 site, for example, becomes phosphorylated early in the disease, and precedes phosphorylation at the pS202/pT205 AT8 site [4], [5].

The phosphorylation of Tau is a reversible process, which implies that the pathological hyperphosphorylation can result from a deregulation of kinase and/or phosphatase activity. Intense research has increased our understanding of the kinases that generate certain phosphorylation events, as well as their complex regulation. Calpain cleavage of the regulatory p35 subunit of CDK5 results in a membrane detachment of the resulting CDK5/p25 complex [6]. Although the intrinsic catalytic activity of this complex towards Tau seems not different from that of CDK5/p35 [7], the cytosolic presence of the CDK5/p25 complex might play a role in Tau's phosphorylation. As a downstream event, T231 phosphorylation by GSK3β requires priming of the S235 site by CDK5 [8], [9], but CDK5 can equally inhibit directly the GSK3β enzyme through phosphorylation of the latter at its S9 [10].

One major brain phosphatase that dephosphorylates phospho-Tau is the Protein Phosphatase 2A (PP2A) [11], [12], [13], a multimeric enzyme consisting at least of a dimeric core enzyme (hereafter abbreviated PP2A_D_) constituted of a catalytic subunit (C subunit) and a scaffolding subunit (A subunit). This heterodimer (PP2A_D_) can further integrate a regulatory B-type subunit to form a heterotrimeric PP2A complex (PP2A_T_) [14], [15]. The particular regulatory B-type subunit, that exhibits itself specific spatial and temporal expression patterns [16], [17], regulates the catalytic activity and specificity of the heterotrimeric PP2A towards the target phospho-protein(s) [18]. One of the most prominent neuronal forms, highly expressed together with the PP2A catalytic C subunit in neurons, is the B-type subunit PR55/Bα, whose crystal structure in the heterotrimeric enzyme (hereafter abbreviated PP2A_T55α_) has recently been determined [19]. A reduced PP2A activity was shown to induce hyperphosphorylation of Tau, including at the pS202/pT205 AT8 site in transgenic mice [20], and has equally been shown in AD at various levels. Firstly, in the hippocampus, a decrease in mRNA expression of the PP2A catalytic C subunit and various B-type subunits has been observed [21]. Secondly, at the protein level, a decreased level of the PR55/Bα subunit due to an increased turnover is associated with the AD pathology [22].

The regulation of PP2A is complex, and involves more than the subunits of the holoenzyme. Polycations can stimulate the activity of PP2A through a direct interaction with the enzyme. As the stimulation is dependent on the type of holoenzyme, the interaction involves the regulatory B-type subunit [23], although an interaction with the substrate has also been reported [24]. An inhibitory interaction was described for α-endosulfine and Arpp-19, two small proteins that after phosphorylation by the Greatwall kinase interact with the PR55/Bδ regulatory subunit, and thereby shut down the activity of PP2A [25], [26]. Another regulatory factor for PP2A activity is Pin1, a phosphorylation dependent prolyl *cis*/*trans* isomerase that targets specifically the pS/pT-P motifs. This isomerase was shown to stimulate the dephosphorylation of Tau by PP2A [27]. Pin1^−/−^ mice show indeed a decreased phosphatase activity towards pS/pT-P motifs and an accumulation of both the pS202/pT205 AT8 and pT231/pS235 AT180 epitopes [28].

Kinases and phosphatases can hence regulate the phosphorylation/dephosphorylation of Tau in a complex and concerted manner, whereby this intricate feedback can *in vivo* control the final phosphorylation level of Tau [29]. Our present work aims to better define the molecular role of PP2A and Pin1 in this process. We first address the question of whether the different phosphorylation sites of Tau are independent with respect to PP2A catalyzed dephosphorylation, or whether the equivalent of a priming mechanism described for certain kinases exists also for some dephosphorylation reactions. Secondly, we investigate the role of Pin1 in stimulating this PP2A-catalyzed dephosphorylation activity towards specific sites. We use Tau *in vitro* phosphorylated by the activated CDK2/CycA3 complex to study the effect of PP2A on its dephosphorylation. We showed previously that CDK2/CycA3 action at the AT180 epitope is equivalent to that of the combined CDK5/p25 and GSK3β kinases and can generate in a robust manner the pS202/pT205 AT8 and pT231/pS235 AT180 epitopes on Tau [30], [31]. We use NMR spectroscopy as an analytical technique that provides a direct and quantitative view on all phosphorylation events in the full length protein [30], [32]. The dephosphorylation reaction directly performed in the NMR tube allows kinetic monitoring of the individual phosphorylation sites in one single experiment. We reveal a subtle regulation of PP2A_T55α_ activity towards the Tau pS202/pT205 AT8 site by the phosphorylation status of T231, whereby phosphorylation of the latter T231 site negatively interferes with the PP2A catalyzed dephosphorylation of pS202. In addition, we find that Pin1 releases this negative feedback between the pT231 and pS202/pT205 AT8 epitopes. Our findings define an additional level of molecular regulation of the PP2A phosphatase towards the phosphorylated Tau protein, whereby the interplay between kinase and phosphatase activity can potentially lead to a stable hyperphosphorylated state of Tau that characterizes AD affected neurons.

## Results

### Specificity of the heterotrimeric PP2A_T55α_ for Pro-directed CDK2/CycA3 phospho-sites of Tau

**Figure 1:**
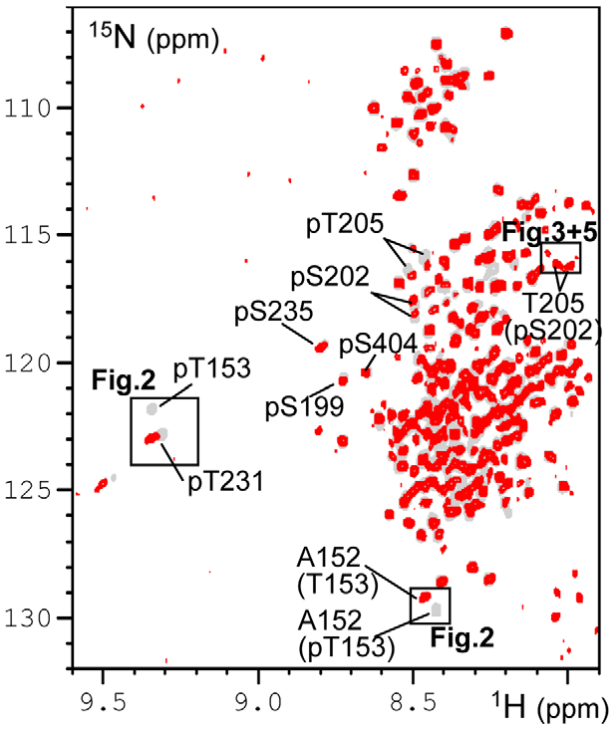
Dephosphorylation of phospho-Tau by the heterotrimeric PP2A_T55α_. Comparison of the [^1^H,^15^N] 2D spectrum of the CDK2/CycA3 phospho-Tau (20 µM, in gray) and same sample incubated 16 hours at 25°C (293K) with 1U of PP2A_T55α_ enzyme (superimposed in red). Cross peaks of the amide function of the corresponding phosphorylated residues are labelled as well as A152(T153 or pT153) and T205(pS202) resonances. Regions of the spectrum enlarged in Fig. 2, 3 and 5 are boxed.

**Figure 2 pone-0021521-g002:**
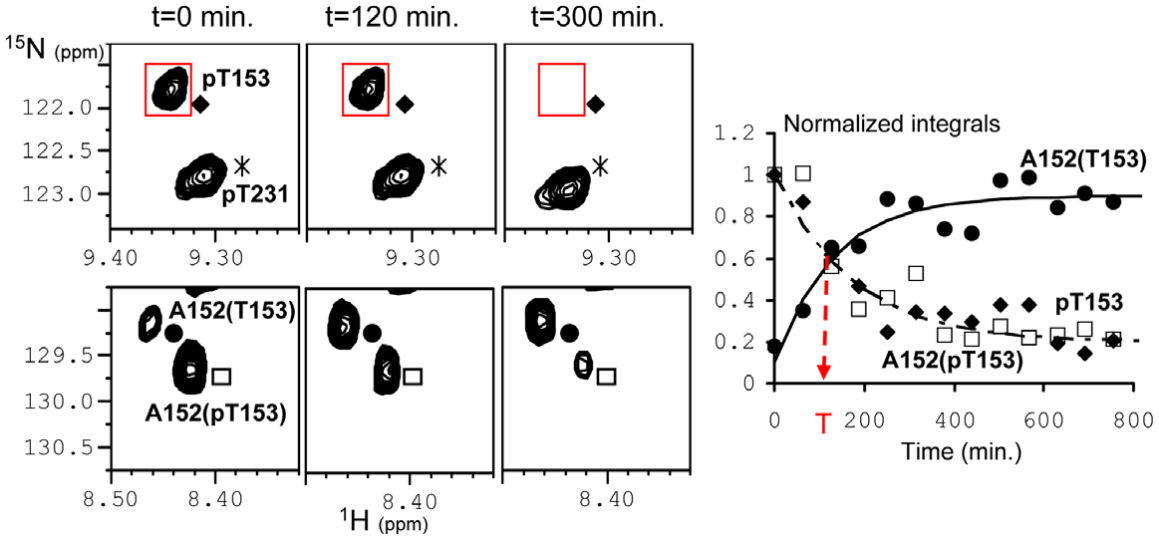
Kinetic of dephosphorylation of phospho-Tau by the heterotrimeric PP2A_T55α_. Panels *from left to right* Control 2D spectrum of phospho-Tau before addition of PP2A_T55α_ (t = 0 min.), second (t = 120 min.) and sixth 2D spectrum (t = 300 min.) in the serie after addition of PP2A_T55α_ (1U). A152(pT153) and A152(T153) stand for the amide resonance of A152 next to a pT153 or a T153 residue in the Tau molecule, respectively. A box is drawn around a peak (see red box around pT153) and is replicated in each spectrum that corresponds to a time-point of the reaction. The surface of the peak inside this box corresponds to an integral value or data-point in the graph on the *right*. This graph shows normalized integral values plotted as a function of time (min.) for pT153 (black diamonds), A152(pT153) (open squares) and A152(T153) (black dots). Data are fitted with mono-exponential curves characterized by the constant T (min.), indicated on the *x-axis* by a red arrow and reported in **Table 1**.

**Figure 3 pone-0021521-g003:**
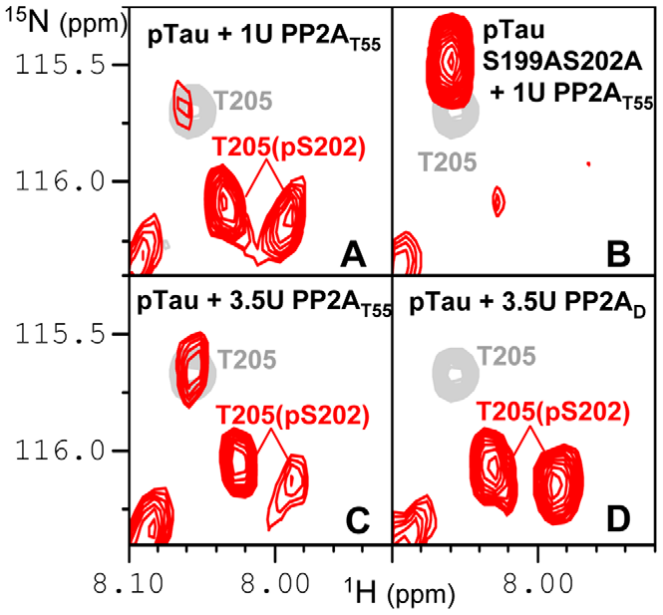
Regulation of the dephosphorylation of the AT8 epitope. Details of the 2D spectra (in red) of phospho-Tau (pTau, A, C, D) and phospho-Tau S202AS199A (pTauS202AS199A, B) after 16 hours of incubation at 25°C (293K) with 1U (A, B) or 3.5U PP2A_T55α_ (C) and 3.5U of PP2A_D_ (D). The spectra are superimposed on the spectrum of the unmodified Tau protein (in gray). T205(pS202) stands for the amide resonance of T205 with a pS202 neighbour in the Tau molecule.

We previously showed that the recombinant CDK2/CycA3 complex generates robust and reproducible phosphorylation of Tau at AD-specific epitopes, with high levels of phosphate incorporation at pS202-pT205 and pT231-pS235 ([30]; **Fig. S1**), recognized respectively by the diagnostic AT8 [1] and AT180 [2] AD-specific monoclonal antibodies. Additional phosphorylation events were observed at position T153 (70–80%), S199 (70–80%) and S404 (50–60%). The heterotrimeric PP2A_T55α_ complex composed of the scaffolding A subunit, the catalytic C subunit and the regulatory PR55/Bα subunit, was added to this CDK2/CycA3-phosphorylated Tau441 sample (phospho-Tau) directly in the NMR tube (**Fig. 1**). Consecutive [^1^H,^15^N] Heteronuclear Single Quantum Correlation spectra (HSQC; hereafter named 2D spectra) yield snapshots of the dephosphorylation reaction with a one hour time resolution, corresponding to the acquisition time of a single 2D spectrum (**Fig. 2**, **Data S1**). Integration of a peak surface for a given resonance in each 2D spectrum during this *in-spectrometer* dephosphorylation reaction gives information on the kinetics of the modification of the corresponding amino acid (**Fig. 2**). Because phosphorylation does not only affect the amide resonance of the modified amino acid but also that of its neighbours, the same reaction can in favourable cases be followed on different cross peaks. The A152 amide peak resonates for example at slightly different frequencies dependent on the phosphorylation status of T153, and the same enzymatic reaction could be followed as the decrease of the pT153 or the A152(pT153) amide cross peaks, but equally as the increase of the A152(T153) amide correlation (**Fig. 2**). Dephosphorylation of pT205 also followed an exponential trend with a similar time constant (**Table 1**) that was best monitored by the unphosphorylated T205 resonance(s) (**Fig. 3**), located in a relatively sparse region of the spectrum [33]. Initially, no peak appears at its exact resonance position in the unphosphorylated Tau protein, but we rather observe two intermediate peaks corresponding to T205(pS202) with a further splitting due to the phosphorylation state of S199 (**Fig. 3A**). This assignment was validated by a dephosphorylation experiment on the phospho-TauS199A/S202A double mutant, where the intermediate forms do not appear, but where we recover immediately the T205 resonance at its position in the unmodified Tau spectrum (**Fig. 3B**). When increasing the phosphatase concentration by a factor of 3.5, a partial dephosphorylation of the complete AT8 epitope on wild type phospho-Tau could be obtained, with 30% of the intensity at the position of T205(S202) after 16 hours of incubation at 25°C (**Fig. 3C**). This latter observation also verifies that we do monitor the enzymatic reaction catalysed by PP2A_T55α_ and not a spontaneous dephosphorylation under our NMR conditions. In addition to the fast dephosphorylation observed for pT153 and pT205 with the phosphate almost completely removed by 3.5 units of PP2A_T55α_ after the first two spectra, two additional classes of residues could be distinguished. The first one contains pT231 and pS404, and is characterized by very slow kinetics with only a marginal reduction of the phospho-resonance after one night. The second group shows intermediate dephosphorylation kinetics, and concerns pS199, pS202 and pS235 (**Fig. 1** **–** [Fig pone-0021521-g002] [Fig pone-0021521-g003] **4** and **Table 1**).

**Figure 4:**
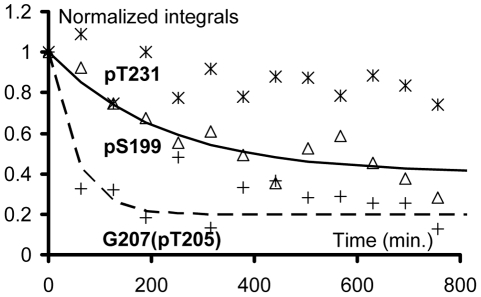
Kinetic of dephosphorylation of phospho-Tau by the heterotrimeric PP2A_T55α_. The dephosphorylation kinetics are obtained by integration of the cross peak of the amide function of the corresponding phospho-residue in the consecutive [^1^H,^15^N] 2D spectra encompassing 60 min. of the *in-spectrometer* reaction at 25°C (293K). The graph shows the differential dephosphorylation by PP2A_T55α_ (3.5 units) of phospho-Tau. Fitting of the data by a mono-exponential curve yielded the time constants T (min.) reported in **Table 1**. pT231 is represented by stars, pS199 by triangles and G207(pT205) by crosses. G207 stands for G207 next to a pT205 in the Tau molecule.

**Table 1:** Specificity of dephosphorylation of the CDK2/CycA3 phospho-sites of Tau by the PP2A_T55α_ and PP2A_D_ phosphatases.

	Tau w.t.			TauT231A
	Trimer (1 U)	Trimer (3.5 U)	Dimer (3.5 U)	Trimer (1 U)
**pT153**	170	60	200	75
**pS199**	240 (30%)	220 (60%)	260 (45%)	200 (40%)
**pS202**	n.d.	300 (25%)	n.d.	200
**pT205**	160	55	120	65
**pT231**	n.d.	n.d.	n.d.	n.d.
**pS235**	n.d.	500 (40%)	n.d.	175 (50%)
**pS404**	n.d.	n.d.	n.d.	n.d.

Integral values of the resonances during a representative dephosphorylation experiment in the spectrometer are fitted with mono-exponential functions of type Integral  =  e^(-t/T)^ or 1-(e^-(t/T)^) with time t and constant T in min, reported in the Table.

Percentage in brackets (): extent of dephosphorylation if not complete.

n.d.: not dephosphorylated.

### Dephosphorylation of phospho-Tau by the PP2A_D_ core enzyme

Several reports have shown the implication of the PR55/Bα regulatory subunit in the substrate anchoring and enzyme efficiency of PP2A_T55α_ [19], [34]. In order to evaluate whether the dimeric enzyme **PP2A_D_**, composed of the A and C subunits without any B-type subunit, would maintain a phosphatase activity, we repeated the same experiments as above with PP2A_D_. For an identical enzyme activity (3.5 units) of PP2A_D_ heterodimer and PP2A_T55α_ heterotrimer as measured with phosphorylase *a* as substrate, the dephosphorylation rate at all phospho-sites of Tau was slower for the PP2A_D_ dimer (**Table 1**). For pT153, we found a three-fold slower rate, and a two-fold slow-down for pT205. Significantly, dephosphorylation of pS202 at the AT8 epitope, such as partially (25%) obtained with 3.5 units of PP2A_T55α_ after 16 hours of incubation at 25°C, did not occur at all with the same amount of PP2A_D_ (**Fig. 3D**).

### Dephosphorylation of the mutant phospho-Tau T231A by PP2A_T55α_

**Figure 5:**
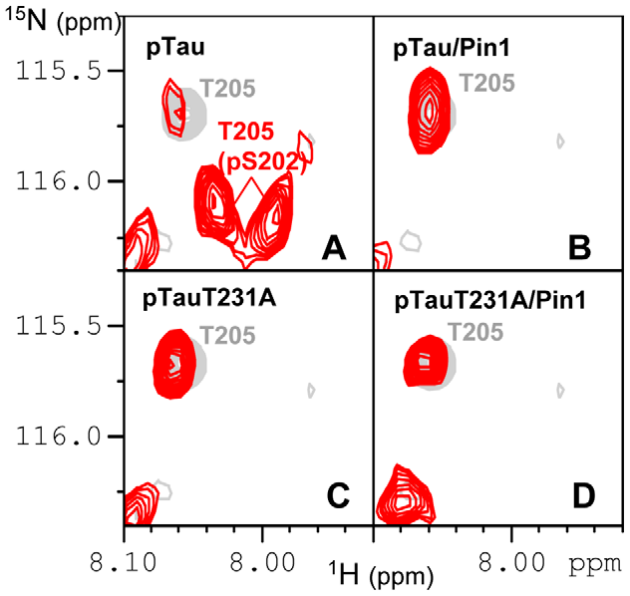
Regulation of the dephosphorylation of the AT8 epitope. Details of the 2D spectrum (in red) of CDK2/CycA3 phospho-Tau (pTau, **A and B**) and phospho-Tau T231A (pTauT231A, **C and D**) after 16 hours of incubation at 25°C (293K) with PP2A_T55α_ (1U) superimposed on the spectrum of the unmodified Tau protein (in gray). pTau and pTauT231A correspond to dephosphorylation without Pin1 (**A and C**) and pTau/Pin1 and pTauT231A/Pin1 with an excess of Pin1 (**B and D**).

The _224_KKVAVVRTPPKSP_236_ peptide of Tau interacts directly with the PR55/Bα subunit, and is able to compete with native Tau for binding to PP2A_T55α_ [34]. Concordantly, the substrate binding groove of PR55/Bα shows a negative character, suggesting a charge-charge interaction between both proteins. Charge-inverting mutations on PR55/Bα indeed led to a decreased level of catalytic efficiency of the holoenzyme [19]. Phosphorylation of the T231 residue would be expected to diminish the positive character of the Tau peptide, and might hence also modulate this interaction and possibly the enzymatic efficiency of the holoenzyme. Measuring the dephosphorylation of a phospho-Tau T231A sample with 1 unit of PP2A_T55α_ phosphatase, we indeed found an increased dephosphorylation rate compared to the wild type phospho-Tau, with a gain of more than two-fold for pT153 and pT205 (**Table 1**). With this low amount of phosphatase, the pS202 position that was not dephosphorylated in the wild type phospho-Tau sample after 16 hours, becomes almost completely dephosphorylated in the phospho-Tau T231A (**Fig. 5**, compare panels A and C). The phosphorylation status of the AT180 epitope hence influences directly the PP2A_T55α_ catalyzed dephosphorylation rate of phospho-Tau and most strikingly of the AT8 epitope.

### Regulation of the PP2A_T55α_ activity by Pin1

Using a cdc2 phosphorylated Tau as substrate, Zhou et al. described a stimulatory effect of Pin1 on the dephosphorylation activity of PP2A_T55α_ [27]. Dephosphorylation of phospho-Tau T231A not being affected by Pin1 led to the hypothesis that the phosphate release assay used in that study monitored the dephosphorylation at this pT231 site, and suggested a regulatory role of Pin1 through its *cis/trans* isomerase activity at this precise pT231-Pro motif [27]. Our result that pT231 is at best marginally dephosphorylated by PP2A_T55α_ (**Fig. 2**, **4**) prompted us to re-examine the precise role of Pin1 in the framework of Tau dephosphorylation by PP2A_T55α_. Because the interaction with Pin1 (and most probably its WW domain, [35]) broadens the NMR signals of the phospho-resonances beyond detection, we monitored the PP2A kinetics through the appearance of the non-phosphorylated forms. The rate of dephosphorylation of pT205 (T = 160 min.) was indeed increased 3 fold by the presence of a threefold excess of Pin1 (T = 55 min.), which is the same ratio of Pin1:phospho-Tau as used in the initial report [27]. Remarkably, pS202, although not dephosphorylated at all by 1 unit of PP2A_T55α_, is completely dephosphorylated in the presence of Pin1 (**Fig. 5**, **compare panels A and B**). This stimulatory effect of Pin1 is however selective, as the dephosphorylation of pT231, monitored by the appearance of the R230(T231) peak, is still negligible.

We next monitored the dephosphorylation of the same phospho-Tau by PP2A_D_ in the absence or presence of Pin1. Although already of reduced activity when compared to PP2A_T55α_ (**Fig. 3**, **compare panels C and D**), the efficiency of the core enzyme slowed down even further upon the addition of Pin1 (**Fig. S2**). We finally tested the effect of Pin1 on the enzymatic efficiency of the PP2A_T55α_ phosphatase complex towards the phospho-Tau T231A mutant. The dephoshorylation of the complete AT8 epitope was faster than in the wild type phospho-Tau (**Fig. 5**, **compare panels A and D**), but was not further stimulated by Pin1 (**Fig. 5**, **compare panels C and D**).

## Discussion

Tau phosphorylation is a reversible process that regulates in a complex manner its physiological role of microtubule stabilization. It is also linked to its pathological role, as the neurofibrillary tangles found inside the neurons of AD patients invariably contain a hyperphosphorylated form of Tau. Analysis with specific antibodies have shown that the latter form is characterized by the simultaneous presence of multiple phosphorylated residues, including the S202/T205 and T231 positions that constitute respectively the AT8 and AT180 epitopes [2]. These phosphorylation events seemingly follow a hierarchical appearance, T231 being one of the earliest sites to be phosphorylated in AD brain before the epitope recognized by the AT8 antibody [4], [36]. In the axon, PP2A_T55α_ is associated with the microtubules [34], and its phosphatase activity counteracts the appearance of this multiply phosphorylated Tau [29]. In addition, it has recently been reported that other neuronal PP2A heterotrimers might indirectly contribute to Tau phosphorylation/dephosphorylation by regulating the activities of GSK3β and CDK5 kinases [37]. However, at a certain stage of the disease, this kinase-phosphatase balance breaks down, and one does observe the accumulation of hyperphosphorylated Tau in the somato-dendritic compartment [29], [38], [39].

In this report, we investigate at the molecular level the enzymatic dephosphorylation of Tau by PP2A_T55α_, the major brain isoform of PP2A directly associated to microtubules and Tau [16], [34], and ask whether certain phosphorylation events on Tau might exert an effect towards its activity at other sites. Our *in vitro* set-up with CDK2/CycA3 kinase generating the AT8 and AT180 epitopes on recombinant ^15^N-labelled Tau [30] and concomitant NMR analysis gives a global view of the Tau phosphorylation pattern as a function of time. We thereby find that PP2A_T55α_ selectively targets certain Tau phosphorylation sites, with fast dephosphorylation of the pT205 and pT153 sites but hardly any for pT231 (**Table 1**). Whereas inefficient dephosphorylation of pT231 was previously described for PHF-Tau [40], our finding of efficient dephosphorylation for the pT153 site has not yet been described in the literature because of the absence of specific antibody. This underscores the advantage of the global view conferred by our NMR approach.

The dimeric PP2A_D_ (PP2A AC) isoform has a lower catalytic efficiency on all Tau phospho-sites compared to the heterotrimeric PP2A_T55α_, emphasizing the crucial role of the B subunit in the function of the PP2A holoenzyme. A correlation between the interaction of Tau with the PR55/Bα subunit and the catalytic efficiency of PP2A_T55α_ was previously described [34], [41], and has recently obtained a molecular basis with the crystal structure of the PP2A_T55α_ holoenzyme [19]. The β-propeller constituting the PR55/Bα subunit indeed contains an acidic groove constituting a potential binding site for the basic _224_KKVAVVRTPPKSP_236_ Tau peptide that can compete with full-length Tau for binding to PP2A [34]. In this mode of interaction, the PR55/Bα regulatory subunit would thus play a role in anchoring the enzyme to its substrate, or alternatively, would exert an allosteric stimulatory effect as observed for protamine or poly-lysine [23]. We did observe that all sites were more rapidly dephosphorylated in the phosphorylated Tau T231A, implying that phosphorylation at the T231 position reduces the PP2A activity. Phosphorylation at this position could modulate the binding mode of the PR55/Bα subunit leading to the inhibition of the PP2A activity. The recent discovery of α-endosulfine or Arpp-19, that both require phosphorylation of S67 by the Greatwall kinase within the already acidic peptide D_66_SGDD_69_ in order to efficiently interact with the PR55/Bδ subunit of the Xenopus mitotic PP2A heterotrimer [26], [25], suggests that different binding modes to the PR55/B regulatory subunits may exist. Phosphorylation of T231, be it by GSK3β after CDK5 priming at the S235 position [8], [9] or by another kinase combination, thus stimulates the phosphorylation at the AT8 epitope via negative feedback on PP2A_T55α_ phosphatase activity. This potentially can lead to a stable state with both epitopes phosphorylated [42], as is found in AD neurons [2].

An additional level of regulation has been described for the phospho-dependent prolyl *cis*/*trans* isomerase Pin1 [43]. Here, we confirm the stimulatory effect of Pin1 on the PP2A_T55α_ catalyzed dephosphorylation of wild type phospho-Tau and its absence in the Tau T231A mutant. Stimulation was most striking for the pS202 site, switching from no dephosphorylation with one unit of PP2A_T55α_ in the absence of Pin1 to complete dephosphorylation in its presence. The effect is however not homogeneous, with at best a weak activity of PP2A_T55α_ towards pT231 despite the presence of Pin1. Moreover, the stimulatory effect directly depends on the PR55/Bα subunit, as Pin1 rather hinders than stimulates the activity of the dimeric core enzyme. Pin1 hence counterbalances the negative regulation of PP2A_T55α_ activity towards the AT8 site following phosphorylation of the AT80 pT231 site, in agreement with the inverse correlation between Pin1 expression and actual neurofibrillary degeneration in AD [28]. Because PP2A_T55α_ has been shown to regulate the phosphorylation status of Tau *in vivo*, a potential trigger for PHF formation might be a shift of the balance between neuronal kinases and phosphatases. Our results show that phosphorylation of T231 directly influences the dephosphorylation rate of pS202, without necessitating an alteration in the PP2A_T55α_ pool. We have reconstituted here the complex system of phosphorylated Tau and the trimeric PP2A_T55α_ phosphatase in the NMR tube with several recombinant proteins, and thereby unravelled a subtle regulatory mechanism. An additional level of regulation has been shown by the prolyl *cis*/*trans* isomerase Pin1. The phosphorylation/dephosphorylation reactions *in vivo* might even be more complex, because of the presence of tubulin and/or other regulatory factors, but even these can be added in the NMR tube [44]. Whereas we previously showed that NMR observation of Tau in live *Xenopus* ovocytes is feasible [45], [46], we believe that both approaches will bring novel insights in the complex regulation of multi-site phosphorylation.

## Materials and Methods

### Expression and purification of Recombinant proteins

Preparation of the recombinant Tau proteins, isotopic enrichment and phosphorylation are described in [30], [32]. The Pin1 protein was expressed as a His Tag fusion from a pET15b plasmid in BL21(DE3) *E. coli* strain [47]. The His Tag was not removed before the assays.

### Preparation of CDK2-CycA3 phospho-Tau

CDK2/CycA3 were prepared as described in [48] except that the bacterial extracts containing recombinant GST-CDK2pT160 (Gluthathion S transferase fusion protein) and CycA3 were mixed before being applied on a Glutathion sepharose FF (5 ml, GE Healthcare). The CDK2/CycA3 complex was recovered from the resin by proteolysis, performed at 4°C overnight with the PreScission protease (GE Healthcare) directly on the Glutathion Sepharose beads in buffer 50 mM Tris pH 8.0, 20 mM NaCl, 1 mM EDTA, 1 mM DTT. The concentration of the complex was evaluated at 280 nm with an extinction coefficient ε of 67420 M^−1^cm^−^1. The complex was kept frozen at −80°C in this buffer until used.

### Purification of dimeric and trimeric PP2A

Purification of PP2A_D_ and PP2A_T55α_ from rabbit skeletal muscle is described in [23]. Calibration of the activity was done using the standard PP2A substrate phosphorylase *a* [23]. One unit is defined as the activity corresponding to removal of 1 nmole/min of ^32^P-phosphate at 30°C.

### NMR sample preparation

NMR buffer was 25 mM Tris-d11, 25 mM NaCl, 2.5 mM EDTA, 1 mM DTT, pH 6.8. Dephosphorylation of 20 µM of CDK2/CycA3 phospho-Tau (or Tau mutants), as defined by absorption at 280 nm, was performed in a volume of 160 µl in 3 mm NMR tubes. Samples with Pin1 are 20 µM phospho-Tau/60 µM Pin1. The PP2A enzyme diluted in 20 µl buffer was directly added in the NMR tube to 140 µl of the phospho-Tau, immediately before starting acquisition of the 2D spectra series.

### NMR spectroscopy

Spectra were acquired at 293K on a 800 MHz equipped with a 3 mm Probe. Parameters of the [^1^H,^15^N]-HSQC (HeteroNuclear Single Quantum Spectroscopy) were 32 scans, 2048 points in the direct dimension and 128 points in the indirect dimension. An optimized d1 of 0.8 s was used. The [^1^H,^15^N]-HSQC are named 2D spectra in the text.

### Data analysis

The peak integrals (**Data S1**) were normalized against an invariant and intense peak in the spectrum corresponding to A85, set to 1 in the integration. Integration was performed with the Topspin 2.1 software (Bruker, Karlsruhe, Germany). Integral data were fitted with a mono-exponential function.

## Supporting Information

Figure S1 **Identification of the phospho-sites of CDK2/CycA3 phospho-Tau.** Superimposition of the [^1^H,^15^N] 2D spectra of the Tau protein (black) and of the CDK2/CycA3 phospho-Tau protein (red). Inset: the enlarged region of the spectrum illustrates phosphorylation of residues S202 and T205.
(PDF)

Figure S2 **Regulation of the dephosphorylation of the pS202/pT205 AT8 epitope.** Details of the 2D spectrum (in red) of CDK2/CycA3 phospho-Tau after 16 hours of incubation at 25°C (293K) with PP2A_D_ (3.5 U) superimposed on the spectrum of the unmodified Tau protein (in gray). **A** dephosphorylation without Pin1 and **B** in presence of an excess of Pin1.
(PDF)

Data S1 **Step by step procedure of NMR data treatment.** **I.** A serie of 2D spectra is acquired, in this example we limited the serie to the first five 2D spectra. The first one in the serie is the control experiment without enzyme and is time zero or starting point in the *in spectrometer* kinetics of dephosphorylation. The following 2D spectra, after the addition of the enzyme, are acquired successively while the NMR tube remains in the spectrometer. This experimental dataset corresponds to the one presented in Fig. 2 and Fig. 4. **II.** Each 2D spectrum required 63 minutes for acquisition. Each data-point is thus obtained every 63 minutes: that is the time resolution in this experiment. **III.** The succession of the 2D spectra will allow to follow the kinetics of the reaction. Duration of each spectrum is cumulated. In this example, we cover with the five 2D spectra 252 minutes of the enzymatic reaction. **IV.** The surface of a peak in a boxed area in the 2D spectra (see point I) or Integrals is calculated by TOPSPIN2.1 Bruker software. This value is directly related to the amount of the corresponding amino acid residue in solution. We follow the evolution of the peak surface or Integral for each amino acid residue of interest (I1 to I4) during the reaction, as detected in each successive spectrum. In our example, I1 corresponds to pT153, I2 to pT231 and I3 to A152. I4 is linked to the resonance of amino acid residue A77. **V.** Data of integration is normalized against integral value of A77 (I4), an invariant and intense peak in the spectra, in order to compensate for any variation of intensity due to potential spectrum to spectrum modification of conditions (for example, addition of the enzyme). **VI.** Data are normalized against the highest integral value of the kinetics for each amino acid residue. In that way, the starting point or ending point of the kinetics is set to one, allowing easier comparison of the evolution of the amount of various amino acid residues during the course of the enzymatic reaction. **VII.** A final table is obtained containing the normalized integral values for each time-point in the kinetics. **VIII.** For each amino acid residue, an exponential curve is fitted to the experimental data by adjusting the value T in the exponential formula e^(-t/T)^. The value T is found by minimizing the sum of the square differences between the calculated and observed data (compare table values in VII and VIII). This constant characterizes the rate of dephosphorylation and is reported in Table 1. A scaling factor (0.8) as well as a residual value depending of the extent of dephosphorylation (0.2 or 0.1) is also used to adjust the formula to the data. Values in the table IX are calculated based on the formula in the *left column* for pT153 (decreasing exponential) and A152 (increasing exponential). **IX.** Data-points from table in VII are graphically represented with the axis being the time-points (each 2D spectrum) and the ordinate the normalized integral value for each time-point (spectrum) reported for several amino acid residue of interest. In this example, pT153 or I1 (open squares) shows a decrease of its initial integral value or peak surface that can be interpreted as a decrease of the corresponding chemical species in solution, in this case due to the dephosphorylation by PP2A. The resonance of A152 (black dots) shows accordingly an increase of its integral value. pT231 integral value remains unaffected throughout the *in spectrometer* enzymatic reaction (stars). The fitted exponential curves are represented by lines.
(PDF)
