# Supplementary figures and images for: Molecular Implication of PP2A and Pin1 in the Alzheimer's Disease Specific Hyperphosphorylation of Tau
Source: PLoS One. 2011 Jun 23;6(6):e21521. doi: 10.1371/journal.pone.0021521 (PMC3121875; doi:10.1371/journal.pone.0021521)

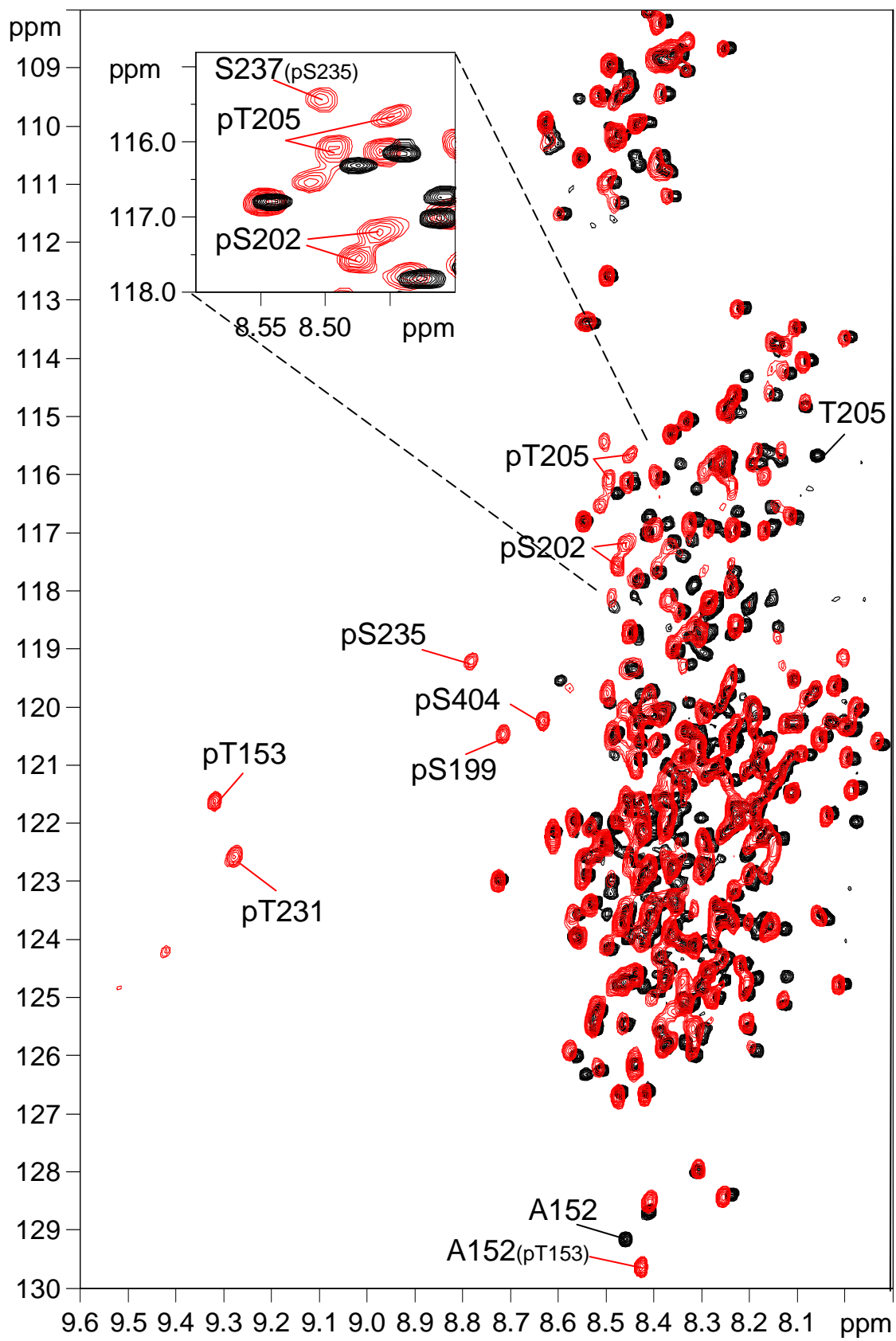

Supplement: Figure S1 — Identification of the phospho-sites of CDK2/CycA3 phospho-Tau. Superimposition of the [1H,15N] 2D spectra of the Tau protein (black) and of the CDK2/CycA3 phospho-Tau protein (red). Inset: the enlarged region of the spectrum illustrates phosphorylation of residues S202 and T205. (PDF) [file pone.0021521.s001.pdf]

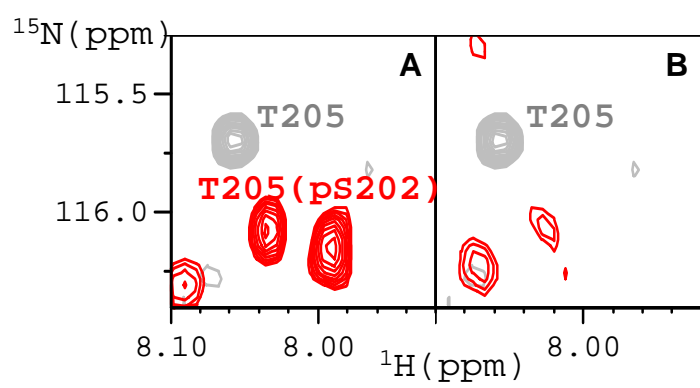

Supplement: Figure S2 — Regulation of the dephosphorylation of the pS202/pT205 AT8 epitope. Details of the 2D spectrum (in red) of CDK2/CycA3 phospho-Tau after 16 hours of incubation at 25°C (293K) with PP2AD (3.5 U) superimposed on the spectrum of the unmodified Tau protein (in gray). A dephosphorylation without Pin1 and B in presence of an excess of Pin1. (PDF) [file pone.0021521.s002.pdf]
