# Supplementary material for: Molecular Implication of PP2A and Pin1 in the Alzheimer's Disease Specific Hyperphosphorylation of Tau
Source: PLoS One. 2011 Jun 23;6(6):e21521. doi: 10.1371/journal.pone.0021521 (PMC3121875; doi:10.1371/journal.pone.0021521)

I

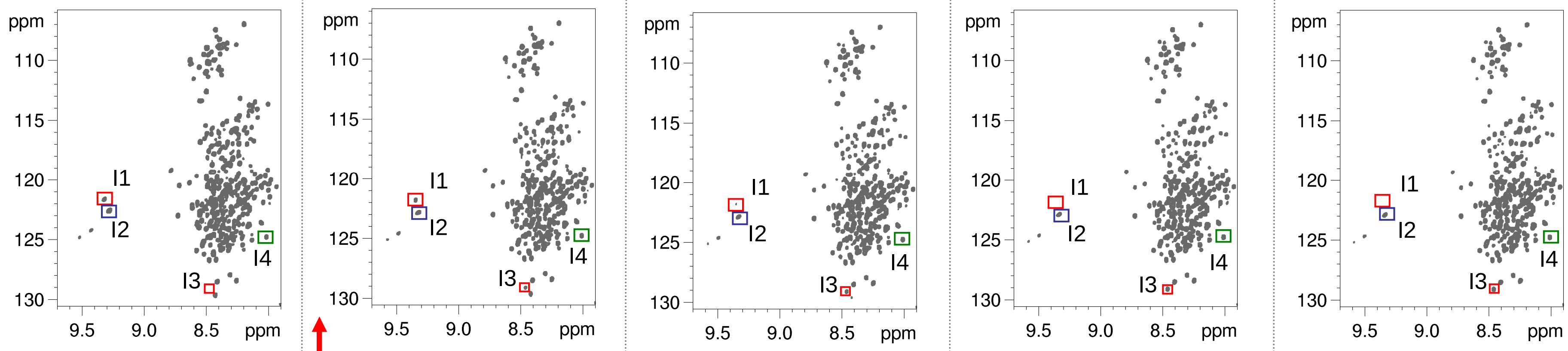

Enzyme addition

II

2D Spectrum Duration

63 min	63 min	63 min	63 min	63 min

III

Cumulative time (Kinetics of the reaction)

t=0 min	t=63 min	t=126 min	t=189 min	t=252 min

Peaks integrals

IV

	t=0 min	t=63 min	t=126 min	t=189 min	t=252 min
I1	2425300	3027300	2359500	2681800	2405900
I2	1799200	1651800	1207400	879960	451840
I3	349210	727590	1406400	1357200	1782700
I4	2902200	3063800	3167300	3053200	2975800

Normalisation on I4

V

	t=0 min	t=63 min	t=126 min	t=189 min	t=252 min
I1	0.84	0.99	0.74	0.88	0.81
I2	0.62	0.54	0.38	0.29	0.15
I3	0.12	0.24	0.44	0.44	0.60

Relative normalisation

VI

	t=0 min	t=63 min	t=126 min	t=189 min	t=252 min
I1	1.00	1.18	0.89	1.05	0.97
I2	1.00	0.87	0.61	0.46	0.24
I3	0.18	0.35	0.65	0.65	0.88

Data summary: first five points of the serie reported in Fig. 2 and 4

		0 min.	63 min.	126 min.	189 min.	252 min.
**I1**	**pT231**	1.00	1.18	0.89	1.05	0.97
**I2**	**pT153**	1.00	0.87	0.46	0.46	0.24
**I3**	**A152**	0.18	0.35	0.65	0.65	0.88

Fitting of the data with an exponential curve

	0 min.	63 min.	126 min.	189 min.	252 min.
**pT153** $0.8*(e^{-(t/170)})+0.2$	1.00	0.75	0.58	0.46	0.38
**A152** $1-(0.8*e^{-(t/150)})-0.1$	0.10	0.37	0.55	0.67	0.75

Graphical summary: first five points of the serie reported in Fig. 2

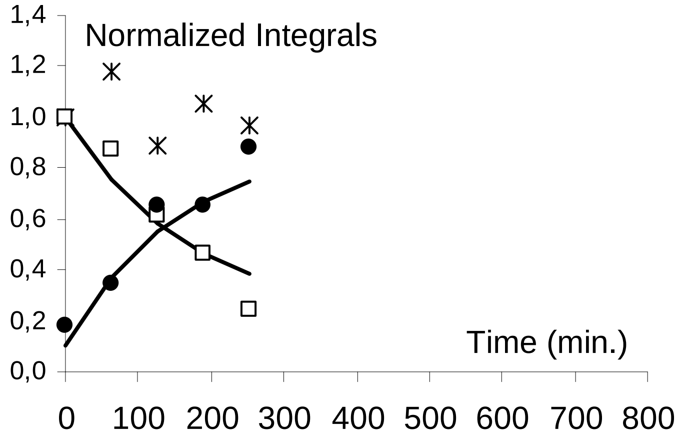

Supplement: Data S1 — Step by step procedure of NMR data treatment. I. A serie of 2D spectra is acquired, in this example we limited the serie to the first five 2D spectra. The first one in the serie is the control experiment without enzyme and is time zero or starting point in the in spectrometer kinetics of dephosphorylation. The following 2D spectra, after the addition of the enzyme, are acquired successively while the NMR tube remains in the spectrometer. This experimental dataset corresponds to the one presented in Fig. 2 and Fig. 4. II. Each 2D spectrum required 63 minutes for acquisition. Each data-point is thus obtained every 63 minutes: that is the time resolution in this experiment. III. The succession of the 2D spectra will allow to follow the kinetics of the reaction. Duration of each spectrum is cumulated. In this example, we cover with the five 2D spectra 252 minutes of the enzymatic reaction. IV. The surface of a peak in a boxed area in the 2D spectra (see point I) or Integrals is calculated by TOPSPIN2.1 Bruker software. This value is directly related to the amount of the corresponding amino acid residue in solution. We follow the evolution of the peak surface or Integral for each amino acid residue of interest (I1 to I4) during the reaction, as detected in each successive spectrum. In our example, I1 corresponds to pT153, I2 to pT231 and I3 to A152. I4 is linked to the resonance of amino acid residue A77. V. Data of integration is normalized against integral value of A77 (I4), an invariant and intense peak in the spectra, in order to compensate for any variation of intensity due to potential spectrum to spectrum modification of conditions (for example, addition of the enzyme). VI. Data are normalized against the highest integral value of the kinetics for each amino acid residue. In that way, the starting point or ending point of the kinetics is set to one, allowing easier comparison of the evolution of the amount of various amino acid residues during the course of the en [file pone.0021521.s003.pdf]
